# Supplementary material for: Effect of gut microbiota on depressive-like behaviors in mice is mediated by the endocannabinoid system
Source: Nat Commun. 2020 Dec 11;11:6363. doi: 10.1038/s41467-020-19931-2 (PMC7732982; doi:10.1038/s41467-020-19931-2)
Supplement: Supplementary file 1 — Supplementary Information [file 41467_2020_19931_MOESM1_ESM.pdf]

SUPPLEMENTARY INFORMATION

Supplementary Table 1: Timeline of the Stressor Exposure used in the Unpredictable Chronic Mild Stress (UCMS) Protocol.

Supplementary Figure 1: Microbiota from UCMS Mice Transfers Depressive-like Behaviors.

Supplementary Figure 2: UCMS Microbiota Transfers Depressive-like Behaviors to Recipient Antibiotic-treated Mice.

Supplementary Figure 3: Complementary Analysis of Metabolomic Profiles in Recipient Mice.

Supplementary Figure 4: Immune Cell Populations, Tryptophan Metabolism and Corticosterone Levels in Recipient Mice

Supplementary Figure 5: Adult Neurogenesis in Dorsal and Ventral Hippocampus are Modulated by the eCB System.

Supplementary Figure 6: Arachidonic Acid or *Lactobacillus plantarum*^{WJL} Complementation Increase Hippocampus Levels of n-3, n-6 PUFA and AEA.

Supplementary Figure 7: Unpredictable Chronic Mild Stress (UCMS) Alters Gut Microbiota and is Transferable to Recipient Mice.

Supplementary Table 1

	Monday	Tuesday	Wednesday	Thursday	Friday
Week 1	Blood & feces (11h-13h)	No sawdust (10h-14h)	3 cage changes (9h30-12h30)	Damp sawdust (10h-13h30)	No sawdust (10h-13h)
	Cage tilt 45° (14h30-19h)	Damp sawdust (14h-18h)	Foreign odor (18h)	Cage tilt 45° (14h-18h)	Foreign odor (14h) Reversal of the L/D cycle (19h)
Week 2	Back to normal L/D cycle (9h)	Damp sawdust (10h-15h)	No sawdust (10h30-15h30)	Cage tilt 45° (10h-15h)	No sawdust (10h-13h)
	Confinement (14h-14h30) Foreign odor(15h)	Cage tilt 45° 18h)	Foreign odor (15h30)	Confinement (15h30-16h) Damp sawdust 16h30)	Foreign odor (15h) Reversal of the L/D cycle (19h)
Week 3	Back to normal L/D cycle (9h)	No sawdust (10h-14h)	No sawdust (10h-14h)	Confinement (10h30-11h)	Damp sawdust (10h-14h)
	Damp sawdust (14h-18h)	Confinement (14h30-15h)	Cage tilt 45° (14h30-19h)	4 cage changes (14h-18h) Foreign odor (18h)	No sawdust (14h-19h) Reversal of the L/D cycle (19h)
Week 4	Back to normal L/D cycle (9h) Cage tilt 45° (9h30-14h)	No sawdust (10h-14h)	3 cage changes (9h30-12h30)	Damp sawdust (10h-15h)	No sawdust (10h-14h)
	Confinement (14h-14h30)	Damp sawdust (14h-18h)	Cage tilt 45° (14h30-18h) Foreign odor (18h)	Confinement (15h30-16h)	Cage tilt 45° (14h30-19h) Reversal of the L/D cycle (19h)

Repeated once for a total of 8 weeks

Supplementary Figure 1

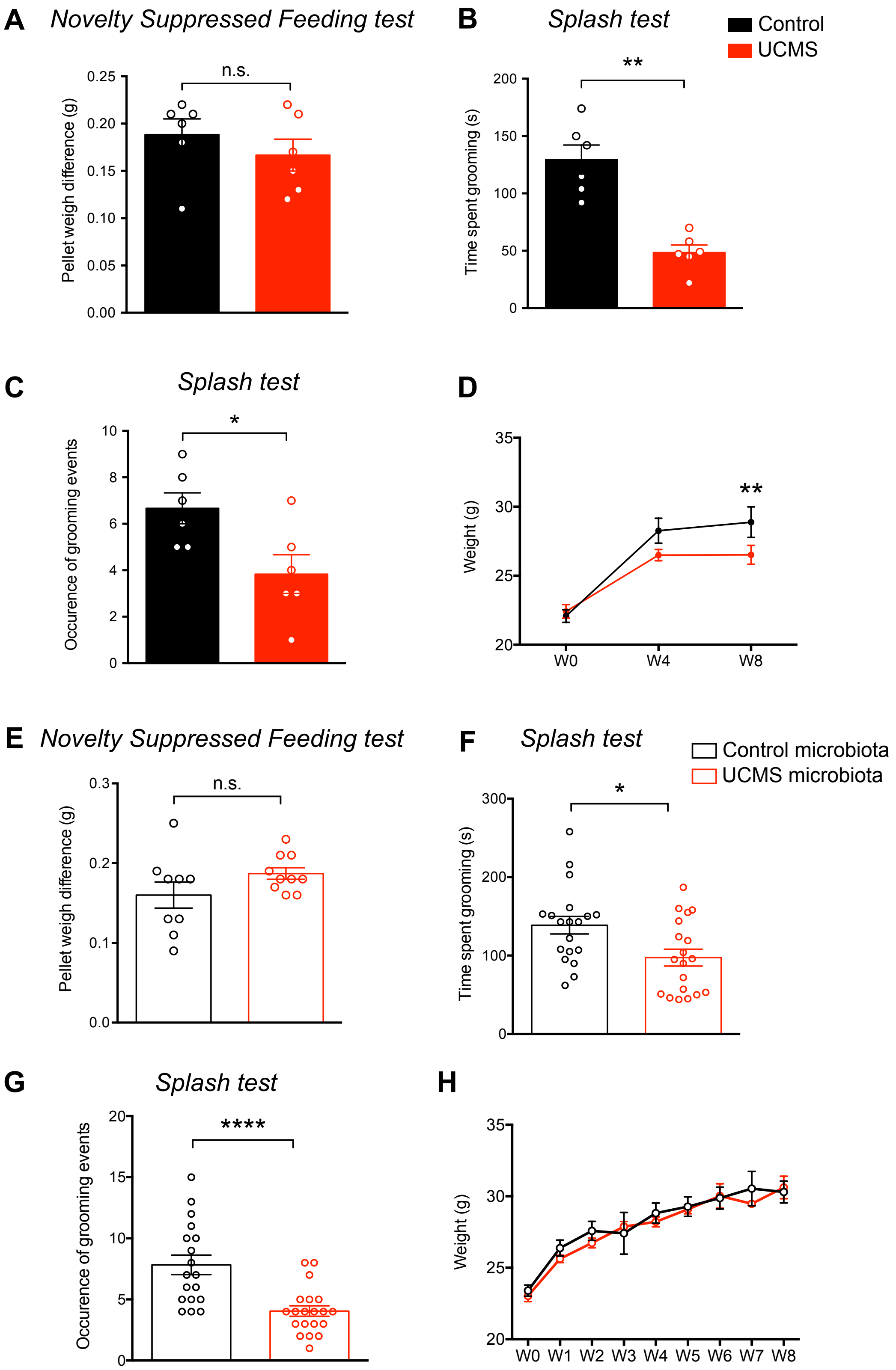

Supplementary Figure 1. Microbiota from UCMS Mice Transfers Depressive-like Behaviors. Control mice, or mice subjected to UCMS (donor mice) (**A-D**), and mice recipient of the microbiota from control or UCMS mice (**E-H**), underwent different behavioral tests. **A**,**E**, Feeding drive of Control mice (n = 6), UCMS mice (n = 6), Control microbiota- (n = 9) and UCMS microbiota-recipient mice (n = 10) in the novelty suppressed feeding test as assessed by the pellet consumption (weight difference) during 10 minutes after the test (Control vs UCMS mice, P = 0.5152; Control microbiota- vs UCMS microbiota-recipient mice, P = 0.2271). **B,F,** Time spent grooming for Control mice (n = 6), UCMS mice (n = 6), Control microbiota- (n = 19) and UCMS microbiota-recipient mice (n = 19) in the splash test (Control vs UCMS mice, P = 0.0022; Control microbiota- vs UCMS microbiota-recipient mice, P = 0.0210). **C,G**, Occurrence of self-grooming events for Control mice (n = 6), UCMS mice (n = 6), Control microbiota- (n = 19 mice) and UCMS microbiota-recipient mice (n = 19 mice) in the splash test (Control vs UCMS mice, P = 0.0325; Control microbiota- vs UCMS microbiota-recipient mice, P < 0.0001). **D, H**, Body weight was measured during the UCMS protocol (n = 6/group, **D**) and after FMT in Control microbiota (n = 9) and UCMS microbiota-recipient mice (n = 10, **H**). Data are represented as mean ± s.e.m. Statistical significance was calculated using the Mann Whitney test (*P < 0.05, ** P < 0.01, *** P < 0.001, **** P < 0.0001, two tailed).

Supplementary Figure 2

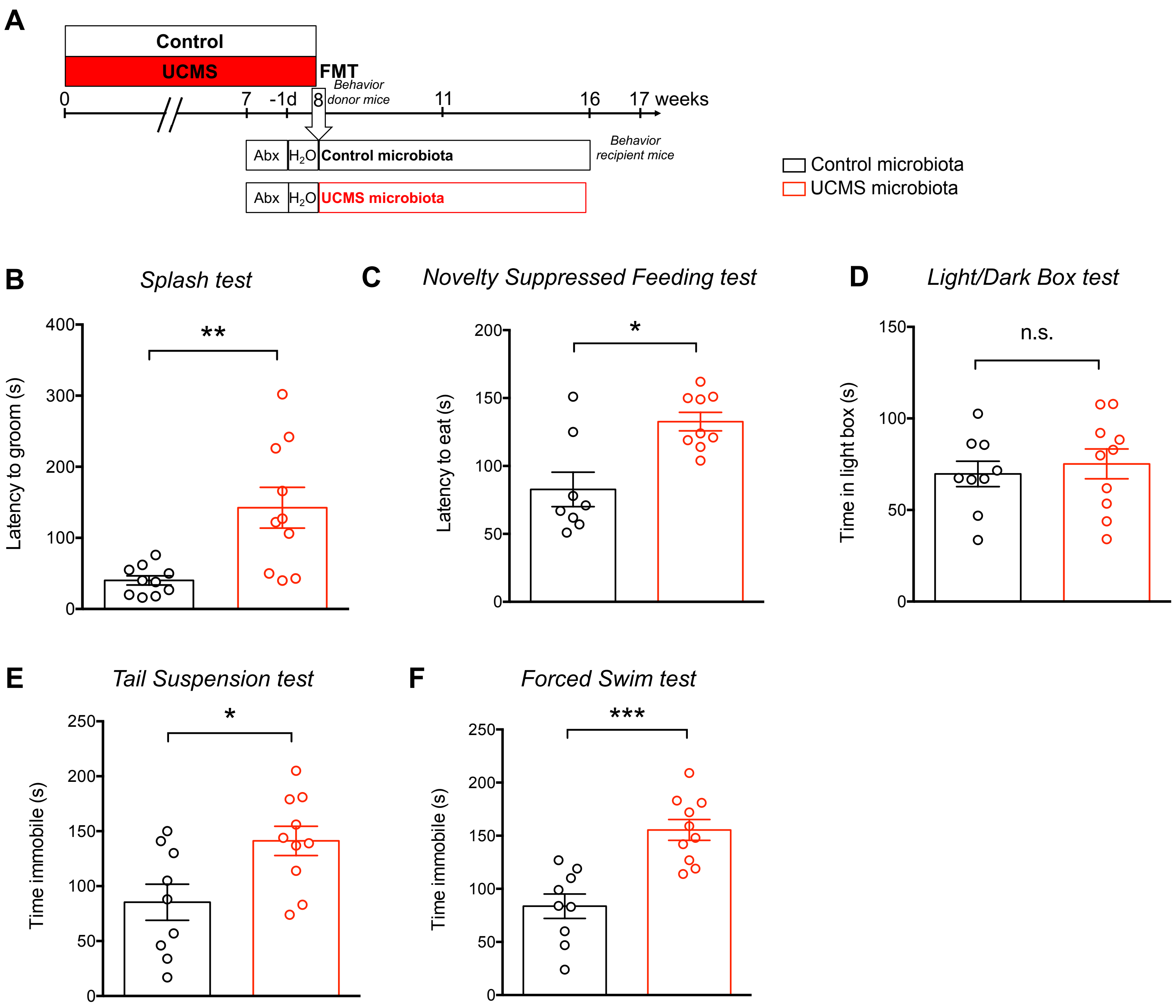

Supplementary Figure 2. Unpredictable Chronic Mild Stress (UCMS) Microbiota Transfers Depressive-like Behaviors to Recipient Antibiotic-treated Mice. **A,** Experimental timeline of Fecal Microbiota Transplantation (FMT) from Control and UCMS mice, respectively 'Control microbiota' and 'UCMS microbiota', to antibiotic (Abx)-treated recipient mice. Recipient mice were given a combination of vancomycin (0.5 g/l), ampicillin (1 g/l), streptomycin (5 g/L), colistin (1 g/l), and metronidazole (0.5 g/l) in their drinking water for 6 consecutive days. Twenty-four hours later, animals were colonized via two rounds of oral gavage with microbiota, separated 3 days apart, and kept in separate sterile isolators. Donor microbiota was acquired from pooled fecal samples from 5-6 animals and resuspended in PBS. **B-F,** Recipient mice underwent different behavioral tests: **B,** Latency to groom in the splash test for Control microbiota- (n = 10) and UCMS microbiota-recipient mice (n = 10) (Control microbiota- vs UCMS microbiota-recipient mice, P = 0.0026). **C,** Latency to eat in a novel environment in the Novelty Suppressed Feeding test for Control microbiota- (n = 8), UCMS microbiota-recipient mice (n = 10) (Control microbiota- vs UCMS microbiota-recipient mice, P = 0.0214). **D,** Time spent in the light box in the Light/Dark Box test for Control microbiota- (n = 9) and UCMS microbiota-recipient mice (n = 10) (Control microbiota- vs UCMS microbiota-recipient mice, P = 0.6038). **E,F**, Time spent immobile in the Tail Suspension test (**E**) and time spent immobile in the Forced Swim Test for Control microbiota- (n = 9,) and UCMS microbiota-recipient mice (n = 10, **F**) (Control microbiota- vs UCMS microbiota-recipient mice, P = 0.0002). Data are represented as mean ± s.e.m. Statistical significance was calculated using the Mann Whitney test (*P < 0.05, ** P < 0.01, *** P < 0.001, two tailed).

Supplementary Figure 3

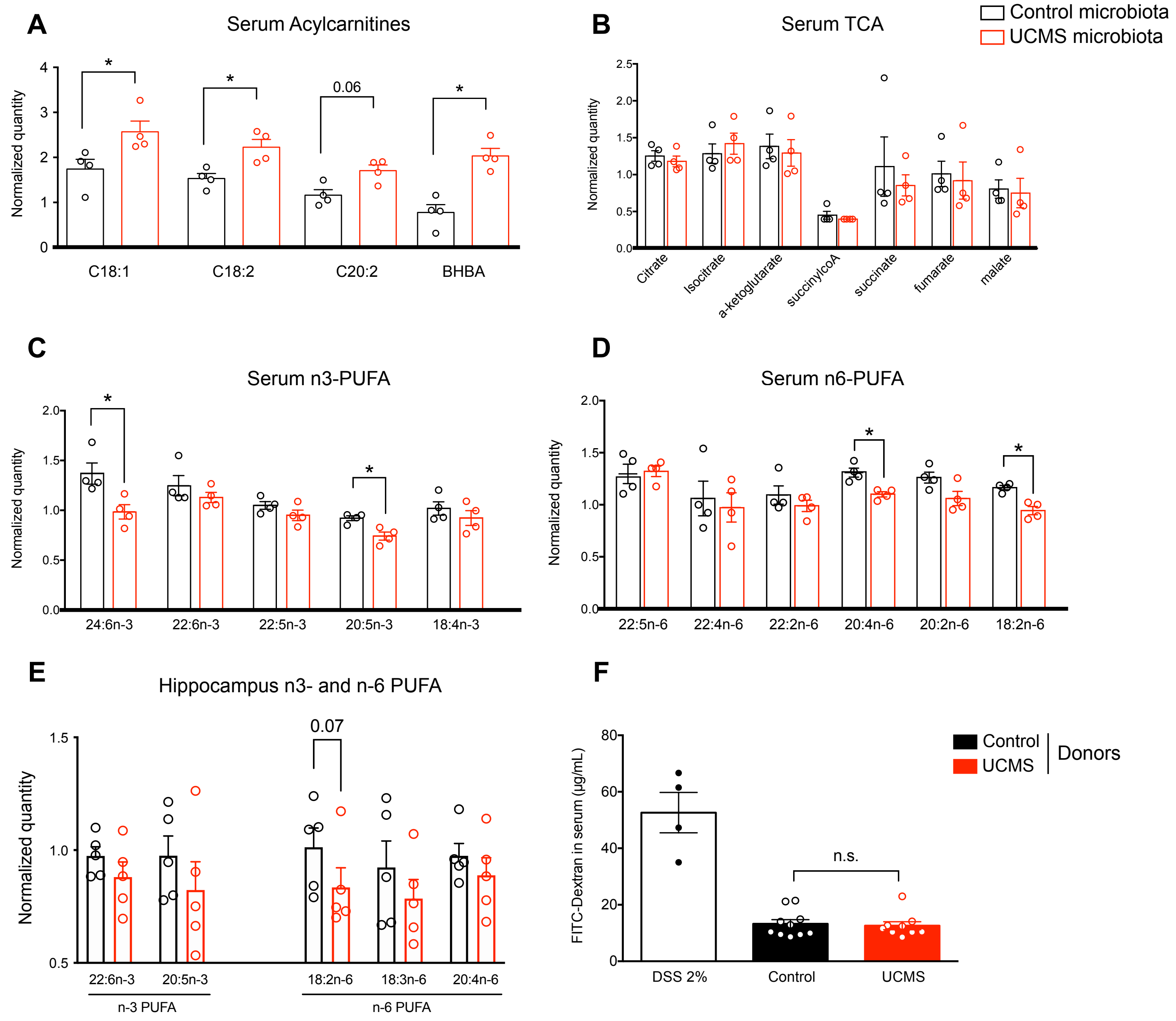

Supplementary Figure 3. Complementary Analysis of Metabolomic Profiles in Recipient Mice. A-D. Normalized levels of serum acylcarnitines (*, P = 0.0286; except for C20:2 with P = 0.0571), (**A**), products of the TCA cycle (**B**), n3-PUFA (*, P = 0.0286) (**C**) and n6-PUFA (*, P = 0.0286) (**D**), (n = 4/group). **E,** Normalized levels of hippocampal n-3 and n-6 PUFA (n = 5/group) (18:2, P = 0.075). Data are represented as mean ± s.e.m. Statistical significance was calculated using the Mann Whitney test (* P < 0.05, two tailed). **F**, Intestinal permeability was measured by FITC intensity in serum after oral gavage with FITC-Dextran in Control (n = 10) and UCMS mice (n = 9). Dextran sodium sulfate (DSS) is used as a positive control (n = 4). Data are represented as mean ± s.e.m. Statistical significance was calculated using the Mann Whitney test (Control vs UCMS mice, P > 0.999, two tailed).

Supplementary Figure 4

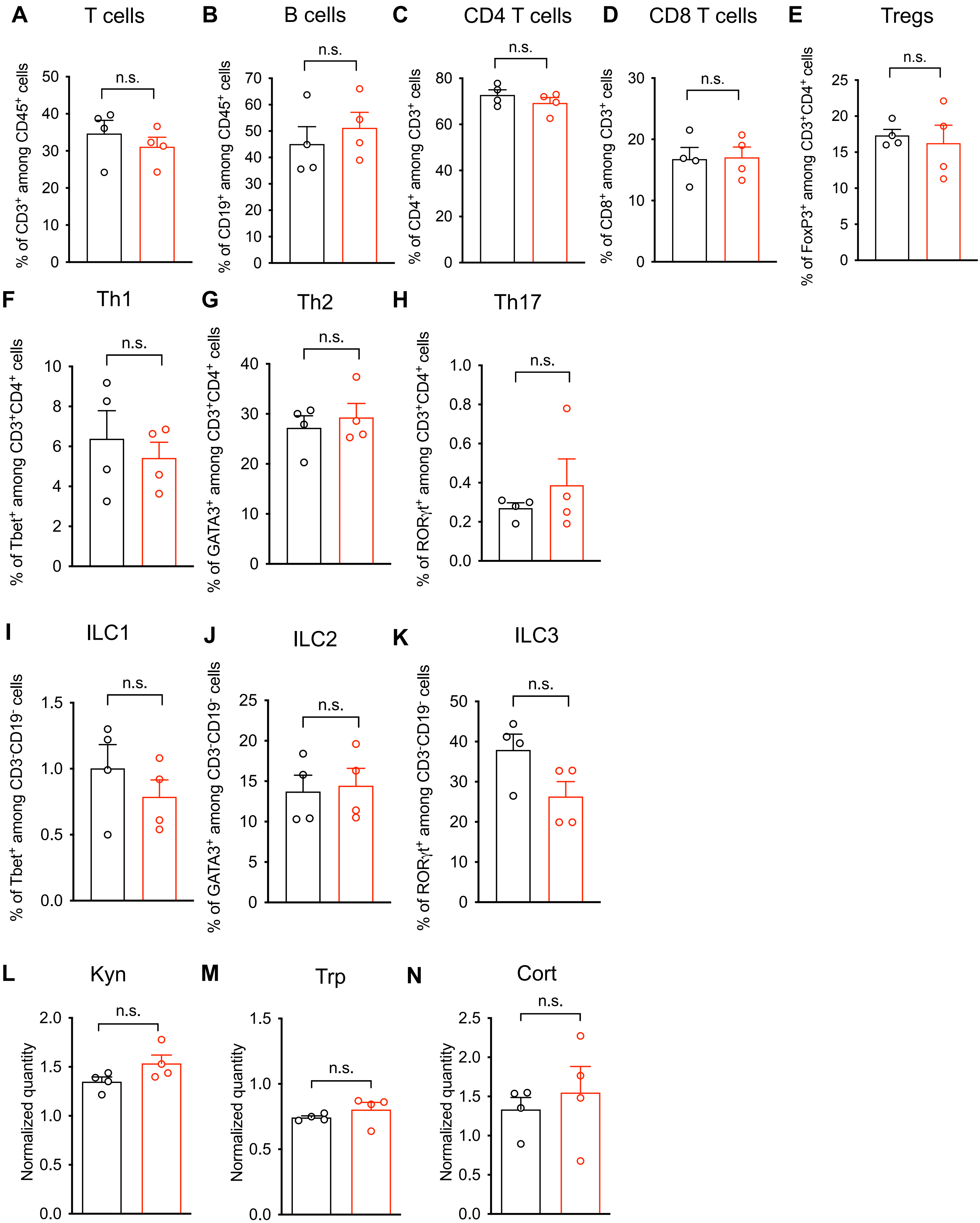

Supplementary Figure 4. Immune Cell Populations, Tryptophan Metabolism and Corticosterone Levels in Recipient Mice. **A**, Percentage of T cells ($CD3^+$) among $CD45^+$ cells; **B**, Percentage of B cells ($CD19^+$) among $CD45^+$ cells; **C**, Percentage of CD4 T cells ($CD4^+$) among T cells ($CD3^+$); **D**, Percentage of CD8 T cells ($CD8^+$) among T cells ($CD3^+$); **E**, Percentage of regulatory T cells (Tregs, $FoxP3^+$) among CD4 T cells ($CD4^+CD3^+$); **F**, Percentage of type 1 CD4 T cells (Th1, $Tbet^+$) among CD4 T cells ($CD4^+CD3^+$); **G**, Percentage of type 2 CD4 T cells (Th2, $GATA3^+$) among CD4 T cells ($CD4^+CD3^+$); **H**, Percentage of type 17 CD4 T cells (Th17, $ROR\gamma t^+$) among CD4 T cells ($CD4^+CD3^+$); **I**, Percentage of type 1 innate lymphoid cells (ILC1, $Tbet^+$) among non-T non-B cells ($CD3^-CD19^-$); **J**, Percentage of type 2 innate lymphoid cells (ILC2, $GATA3^+$) among non-T non-B cells ($CD3^-CD19^-$); **K**, Percentage of type 3 innate lymphoid cells (ILC3, $ROR\gamma t^+$) among non-T non-B cells ($CD3^-CD19^-$) in the lamina propria of control microbiota (n = 4) and UCMS microbiota recipient mice (n = 4). **L**, Normalized levels of kynurenin; **M**, Normalized levels of tryptophan, **N**, Normalized levels of corticosterone in the serum of control microbiota (n = 4) and UCMS microbiota recipient mice (n = 4). Data are represented as mean ± s.e.m. Statistical significance was calculated using the Mann Whitney test (two tailed).

Supplementary Figure 5

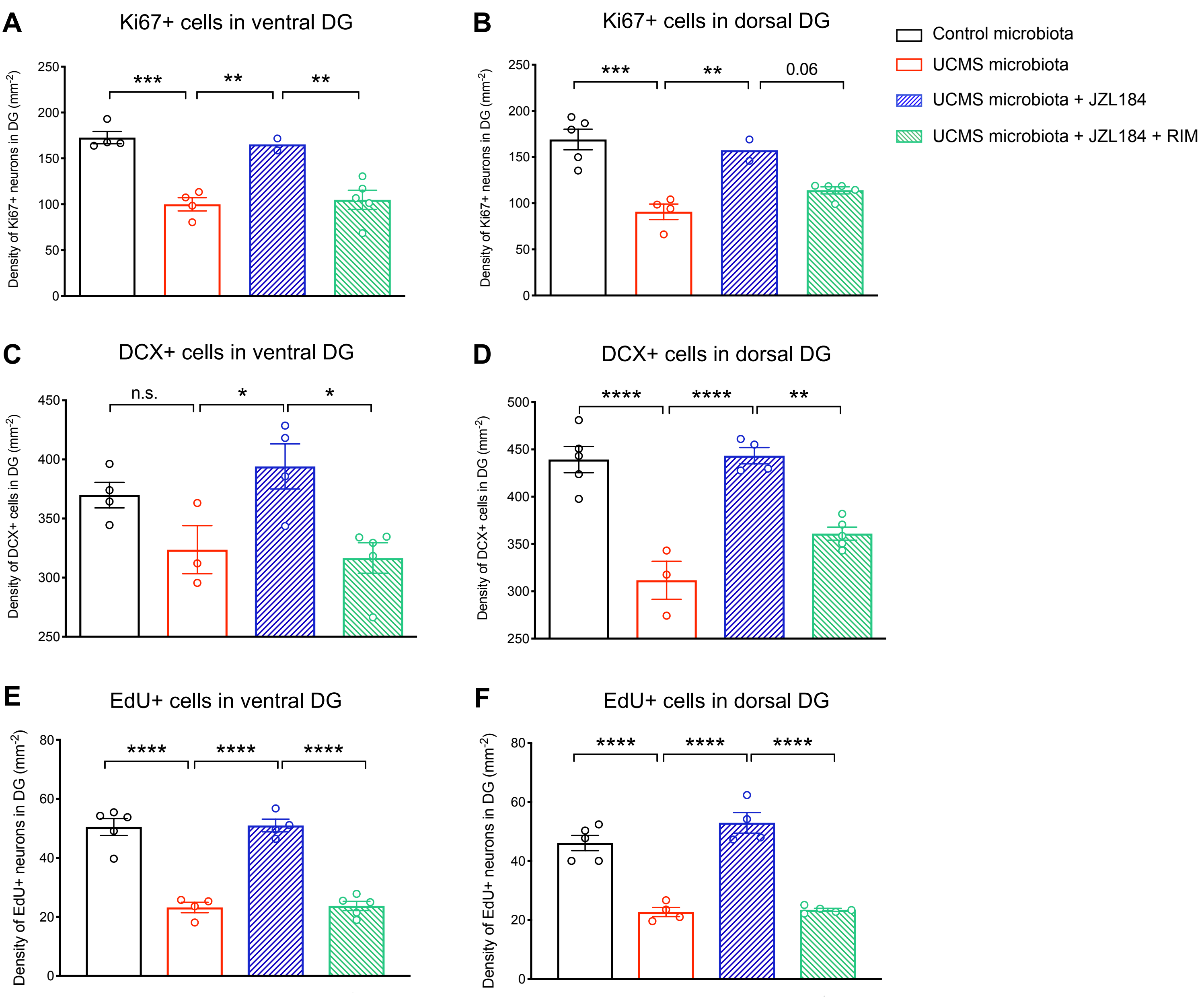

Supplementary Figure 5. Adult Neurogenesis in Dorsal and Ventral Hippocampus are Modulated by the eCB System. A, Quantitative evaluation of the density of Ki67$^+$ cells in ventral DG for Control microbiota-recipient mice (*n* = 4), UCMS microbiota-recipient mice (*n* = 4), UCMS microbiota-recipient mice treated with JZL184 (*n* = 2) and UCMS microbiota-recipient mice treated with JZL184 and rimonabant (*n* = 5). (Control microbiota- vs UCMS microbiota-recipient mice, *P* = 0.0005; UCMS microbiota-recipient mice vs UCMS microbiota-recipient mice + JZL184, *P* = 0.006; UCMS microbiota-recipient mice + JZL184 vs UCMS microbiota-recipient mice + JZL184 + RIM, *P* = 0.0081). **B**, Quantitative evaluation of the density of Ki67$^+$ cells in dorsal DG for Control microbiota-recipient mice (*n* = 5), UCMS microbiota-recipient mice (*n* = 4), UCMS microbiota-recipient mice treated with JZL184 (*n* = 2), UCMS microbiota-recipient mice treated with JZL184 and rimonabant (*n* = 5). (Control microbiota- vs UCMS microbiota-recipient mice, *P* = 0.0002; UCMS microbiota-recipient mice vs UCMS microbiota-recipient mice + JZL184, *P* = 0.0053; UCMS microbiota-recipient mice + JZL184 vs UCMS microbiota-recipient mice + JZL184 + RIM, *P* = 0.0586). **C**, Quantitative evaluation of the density of DCX$^+$ cells in ventral DG for Control microbiota-recipient mice (*n* = 4), UCMS microbiota-recipient mice (*n* = 3), UCMS microbiota-recipient mice treated with JZL184 (*n* = 4), UCMS microbiota-recipient mice treated with JZL184 and rimonabant (*n* = 5). Control microbiota- vs UCMS microbiota-recipient mice, *P* = 0.2603; UCMS microbiota-recipient mice vs UCMS microbiota-recipient mice + JZL184, *P* = 0.0498; UCMS microbiota-recipient mice + JZL184 vs UCMS microbiota-recipient mice + JZL184 + RIM, *P* = 0.0135. **D**, Quantitative evaluation of the density of DCX$^+$ cells in dorsal DG for Control microbiota-recipient mice (*n* = 5), UCMS microbiota-recipient mice (*n* = 3), UCMS microbiota-recipient mice treated with JZL184 (*n* = 4) and UCMS microbiota-recipient mice treated

with JZL184 and rimonabant ($n = 5$). Control microbiota- vs UCMS microbiota-recipient mice, $P < 0.0001$; UCMS microbiota-recipient mice vs UCMS microbiota-recipient mice + JZL184, $P < 0.0001$; UCMS microbiota-recipient mice + JZL184 vs UCMS microbiota-recipient mice + JZL184 + RIM, $P = 0.0014$. **E**, Quantitative evaluation of the density of EdU^+ cells in ventral DG for Control microbiota-recipient mice ($n = 5$), UCMS microbiota-recipient mice ($n = 4$), UCMS microbiota-recipient mice treated with JZL184 ($n = 4$), UCMS microbiota-recipient mice treated with JZL184 and rimonabant ($n = 5$). Control microbiota- vs UCMS microbiota-recipient mice, $P < 0.0001$; UCMS microbiota-recipient mice vs UCMS microbiota-recipient mice + JZL184, $P < 0.0001$; UCMS microbiota-recipient mice + JZL184 vs UCMS microbiota-recipient mice + JZL184 + RIM, $P < 0.0001$. **F,** Quantitative evaluation of the density of EdU^+ cells in dorsal DG for Control microbiota-recipient mice ($n = 5$), UCMS microbiota-recipient mice ($n = 4$), UCMS microbiota-recipient mice treated with JZL184 ($n = 4$) and UCMS microbiota-recipient mice treated with JZL184 and rimonabant ($n = 5$). Control microbiota- vs UCMS microbiota-recipient mice, $P < 0.0001$; UCMS microbiota-recipient mice vs UCMS microbiota-recipient mice + JZL184, $P < 0.0001$; UCMS microbiota-recipient mice + JZL184 vs UCMS microbiota-recipient mice + JZL184 + RIM, $P < 0.0001$. Scale bars: 100µm. Data are represented as mean ± s.e.m. Statistical significance was calculated using One-way ANOVA with Tukey's multiple comparisons test (*$P < 0.05$, ** $P < 0.01$, *** $P < 0.005$, **** $P < 0.0001$).

Supplementary Figure 6

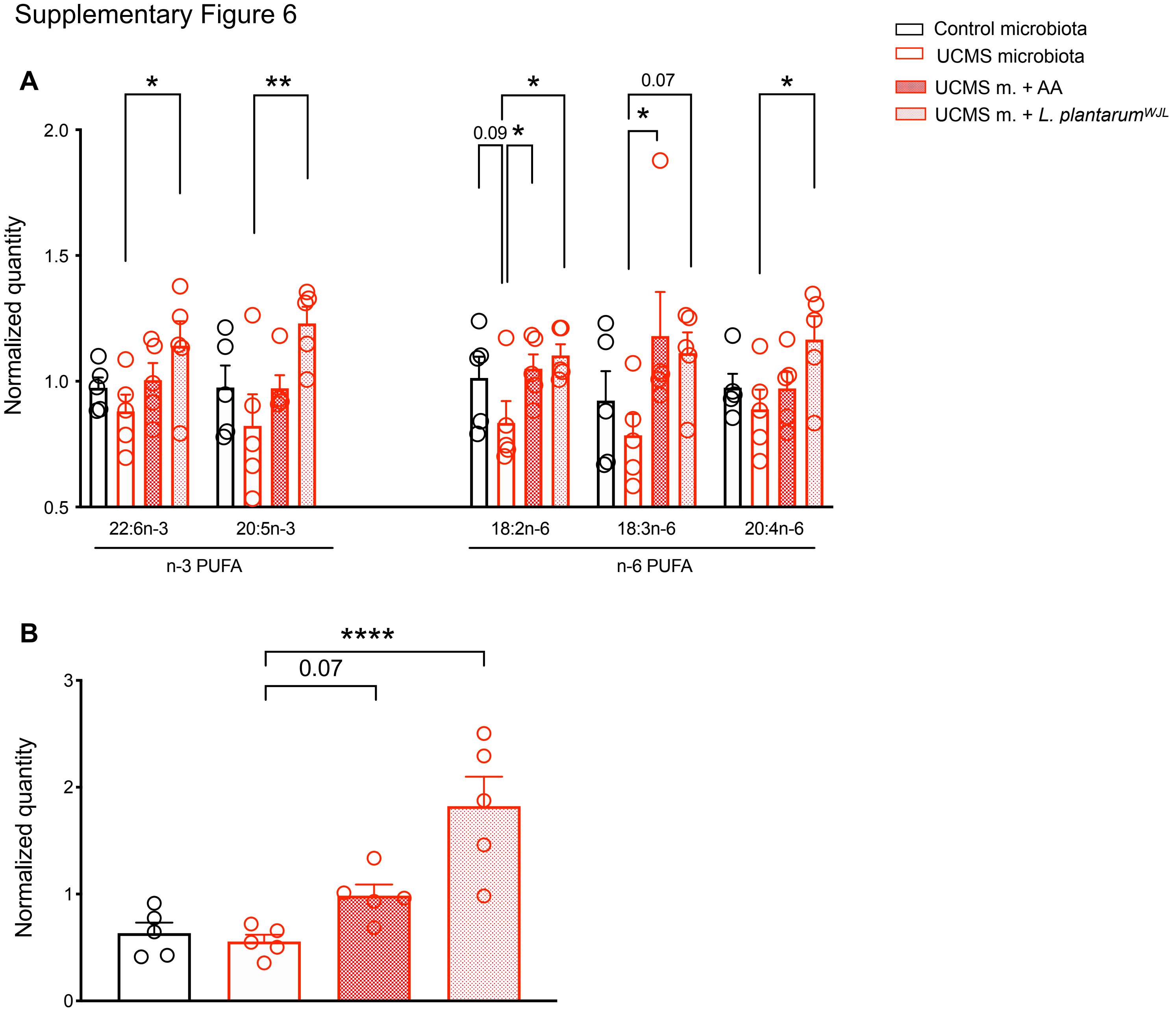

Supplementary Figure 6. Arachidonic Acid or *L. plantarum*^{WJL} Complementation Increase Hippocampus Levels of n-3, n-6 PUFA and AEA. A, Normalized levels of n-3 and n-6 PUFA in the hippocampus of recipient mice, supplemented with either AA or *L. plantarum*^{WJL} (n=5/group), as determined by LC-MS. Control microbiota and UCMS microbiota are the same as in supplementary figure 3E. Data are represented as mean± s.e.m. Statistical significance was calculated using One-way ANOVA test for each selected PUFA (* P<0.05, ** P<0.01). (C22:6, UCMS microbiota vs UCMS microbiota + *Lp*^{WJL}, P = 0.019; C20:5, UCMS microbiota vs UCMS microbiota + *Lp*^{WJL}, P = 0.005; C18:2, Control microbiota vs UCMS microbiota, P = 0.09; UCMS microbiota vs UCMS microbiota + AA, P = 0.047; UCMS microbiota vs UCMS microbiota + *Lp*^{WJL}, P = 0.016; C18:3, UCMS microbiota vs UCMS microbiota + AA, P = 0.035; UCMS microbiota vs UCMS microbiota + *Lp*^{WJL}, P = 0.074; UCMS microbiota vs UCMS microbiota + *Lp*^{WJL}, P = 0.018). **B**, Normalized levels of anandamide (AEA) in the hippocampus of recipient mice, supplemented with either AA or *L. plantarum*^{WJL} (n=5/group), as determined by LC-MS. Control microbiota and UCMS microbiota are the same as in figure 2F. Data are represented as mean ± s.e.m. Statistical significance was calculated using One-way ANOVA test (* P<0.05, **** P<0.0001). (UCMS microbiota vs UCMS microbiota + AA, P = 0.075; UCMS microbiota vs UCMS microbiota + *Lp*^{WJL}, P < 0.0001).

Supplementary Figure 7

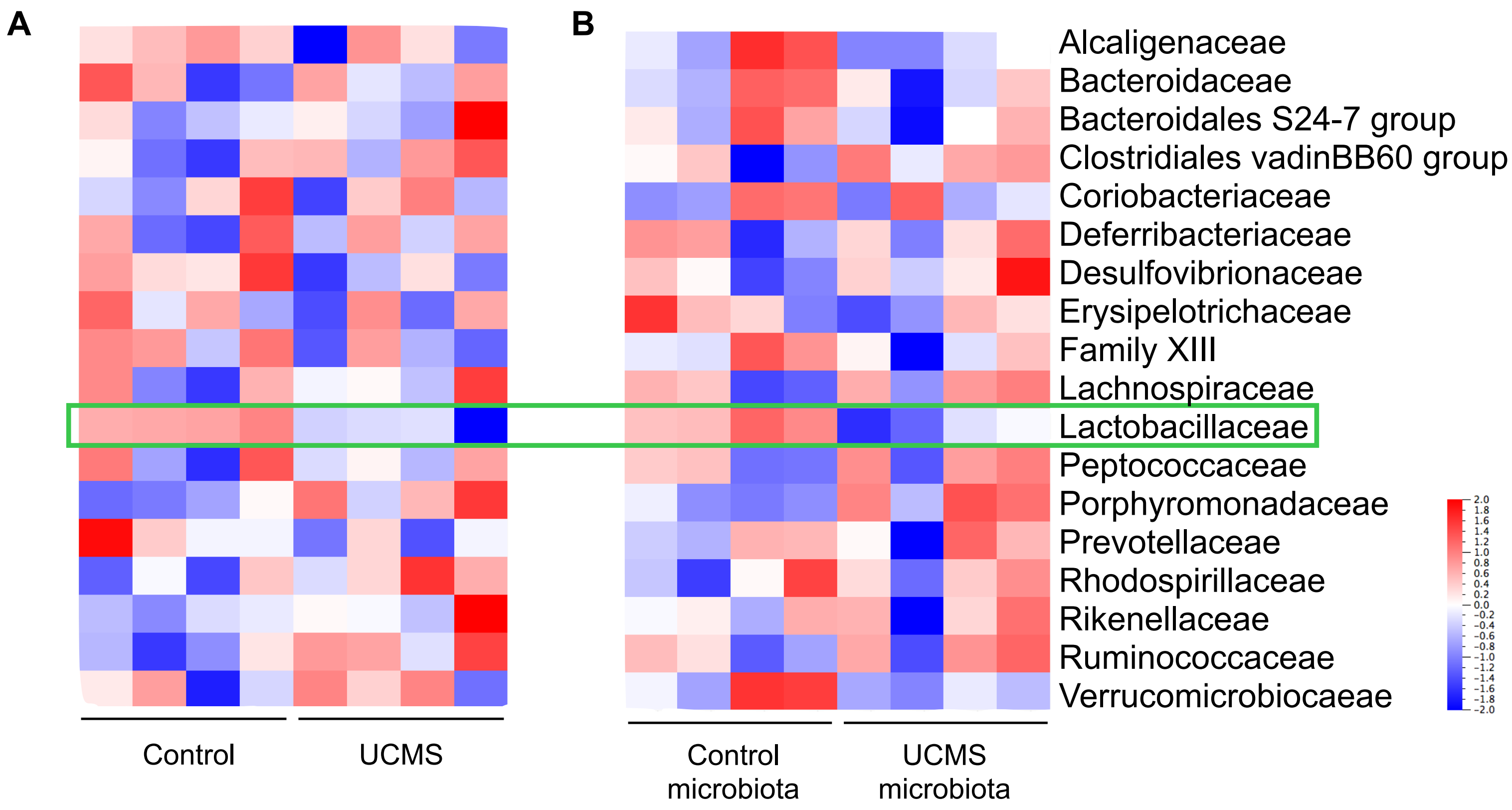

Supplementary Figure 7 Unpredictable Chronic Mild Stress (UCMS) Alters Gut Microbiota and is Transferable to Recipient Mice. The 16S rDNA of the intestinal microbiota was sequenced and analyzed at the level of bacterial families in donor (*n* = 4/group) (**A**) and recipient mice (*n* = 4/group) (**B**).
