# Supplementary material for: Effect of gut microbiota on depressive-like behaviors in mice is mediated by the endocannabinoid system
Source: Nat Commun. 2020 Dec 11;11:6363. doi: 10.1038/s41467-020-19931-2 (PMC7732982; doi:10.1038/s41467-020-19931-2)
Supplement: Supplementary file 3 — Reporting Summary [file 41467_2020_19931_MOESM3_ESM.pdf]

nature research

Corresponding author(s): Lledo Pierre-Marie

Last updated by author(s): Oct 23, 2020

Reporting Summary

Nature Research wishes to improve the reproducibility of the work that we publish. This form provides structure for consistency and transparency in reporting. For further information on Nature Research policies, see our Editorial Policies and the Editorial Policy Checklist.

Statistics

For all statistical analyses, confirm that the following items are present in the figure legend, table legend, main text, or Methods section.

n/a	Confirmed	
☐	☒	The exact sample size (n) for each experimental group/condition, given as a discrete number and unit of measurement
☐	☒	A statement on whether measurements were taken from distinct samples or whether the same sample was measured repeatedly
☐	☒	The statistical test(s) used AND whether they are one- or two-sided<br>*Only common tests should be described solely by name; describe more complex techniques in the Methods section.*
☒	☐	A description of all covariates tested
☐	☒	A description of any assumptions or corrections, such as tests of normality and adjustment for multiple comparisons
☐	☒	A full description of the statistical parameters including central tendency (e.g. means) or other basic estimates (e.g. regression coefficient) AND variation (e.g. standard deviation) or associated estimates of uncertainty (e.g. confidence intervals)
☐	☒	For null hypothesis testing, the test statistic (e.g. F, t, r) with confidence intervals, effect sizes, degrees of freedom and P value noted<br>*Give P values as exact values whenever suitable.*
☒	☐	For Bayesian analysis, information on the choice of priors and Markov chain Monte Carlo settings
☒	☐	For hierarchical and complex designs, identification of the appropriate level for tests and full reporting of outcomes
☒	☐	Estimates of effect sizes (e.g. Cohen's d, Pearson's r), indicating how they were calculated

Our web collection on statistics for biologists contains articles on many of the points above.

Software and code

Policy information about availability of computer code

Data collection	N/A
Data analysis	All statistical analyses were performed using GraphPad Prism v6, Qlucore Omics Explorer, General: Microsoft Excel v15.33

For manuscripts utilizing custom algorithms or software that are central to the research but not yet described in published literature, software must be made available to editors and reviewers. We strongly encourage code deposition in a community repository (e.g. GitHub). See the Nature Research guidelines for submitting code & software for further information.

Data

Policy information about availability of data

All manuscripts must include a data availability statement. This statement should provide the following information, where applicable:

- Accession codes, unique identifiers, or web links for publicly available datasets
- A list of figures that have associated raw data
- A description of any restrictions on data availability

The metabolomics datasets of mouse serum can be accessed at MetaboLights [https://www.ebi.ac.uk/metabolights/index] (Project ID: MTBLS121). The metabolomics datasets of mouse hippocampus tissues can also be accessed at MetaboLights [https://www.ebi.ac.uk/metabolights/index] (Project ID: MTBLS2099). A reporting summary for this article is available as a Supplementary Information file. All other data supporting the findings of this study are available from the corresponding author on reasonable request.

Field-specific reporting

Please select the one below that is the best fit for your research. If you are not sure, read the appropriate sections before making your selection.

- [x] Life sciences
- [ ] Behavioural & social sciences
- [ ] Ecological, evolutionary & environmental sciences

For a reference copy of the document with all sections, see nature.com/documents/nr-reporting-summary-flat.pdf

Life sciences study design

All studies must disclose on these points even when the disclosure is negative.

Sample size	No statistical methods were used to predetermine sample size. Sample size was chosen based on the magnitude and consistency of measurable differences between groups, application of standard practices within the field and economical constraints.<br>Whenever possible, experimental group sizes of at least 4 animals were used. In some cases, experimental groups of 3 animals were used. For all findings, at least two independent experiments were conducted.
Data exclusions	In some occasions, DNA from fecal material did not yield sufficient DNA 16S amplicon or sequencing reads.
Replication	Behavioral data were reproduced by independent experimenters.<br>All experimental findings described in this manuscript were reliably reproduced.
Randomization	All behavioral tests were performed by randomizing order of passage between groups.
Blinding	All analysis were performed blind except for the splash test and the novelty suppressed feeding test that required direct human assessment.

Reporting for specific materials, systems and methods

We require information from authors about some types of materials, experimental systems and methods used in many studies. Here, indicate whether each material, system or method listed is relevant to your study. If you are not sure if a list item applies to your research, read the appropriate section before selecting a response.

Materials & experimental systems

n/a	Involved in the study	
[ ]	[x]	Antibodies
[x]	[ ]	Eukaryotic cell lines
[x]	[ ]	Palaeontology and archaeology
[ ]	[x]	Animals and other organisms
[x]	[ ]	Human research participants
[x]	[ ]	Clinical data
[x]	[ ]	Dual use research of concern

Methods

n/a	Involved in the study	
[x]	[ ]	ChIP-seq
[x]	[ ]	Flow cytometry
[x]	[ ]	MRI-based neuroimaging

Antibodies

Antibodies used	Rabbit anti-DCX Abcam Cat#ab18723<br>Rabbit anti-Ki67 Abcam Cat#ab16667<br>Mouse anti-NeuN Millipore Cat#MAB377<br>Goat anti-rabbit Alexa Fluor 555 Molecular Probes Cat#A32732<br>p-mTOR (S2448) Cell Signaling Cat#2971<br>mTOR Cell Signaling Cat#2972<br>p-p70S6K (T389) Cell Signaling Cat#9205<br>P70S6K Cell Signaling Cat#9202<br>p-rpS6 (S235/236) Cell Signaling Cat#2211<br>rpS6 Cell Signaling Cat#2217<br>GAPDH Cell Signaling Cat#2118<br>Anti-rabbit IgG, HRP-linked antibody Cell Signaling Cat#7074
Validation	Antibodies were chosen based on the available literature. All staining reproduced previously published results or data available from the manufacturers.

Animals and other organisms

Policy information about studies involving animals; ARRIVE guidelines recommended for reporting animal research

Laboratory animals	Adult male C57BL/6J mice (8-10 weeks old) were purchased from Janvier laboratories (St Berthevin, France) and maintained under specific-pathogen free (SPF) conditions at the Institut Pasteur animal care facility. Germ-free C57BL/6J mice were generated at the Gnotobiology Platform of the Institut Pasteur and routinely monitored for sterility.<br>Mice were provided with food and water ad libitum and housed under a strict 12 h light-dark cycle. All animal experiments were approved by the committee on animal experimentation of the Institut Pasteur and by the French Ministry of Research.
Wild animals	*Provide details on animals observed in or captured in the field; report species, sex and age where possible. Describe how animals were caught and transported and what happened to captive animals after the study (if killed, explain why and describe method; if released, say where and when) OR state that the study did not involve wild animals.*
Field-collected samples	*For laboratory work with field-collected samples, describe all relevant parameters such as housing, maintenance, temperature, photoperiod and end-of-experiment protocol OR state that the study did not involve samples collected from the field.*
Ethics oversight	Institut Pasteur and French Ministry of Research DAP200025

Note that full information on the approval of the study protocol must also be provided in the manuscript.
